# A review of imaging techniques for systems biology

**DOI:** 10.1186/1752-0509-2-74

**Published:** 2008-08-12

**Authors:** Armen R Kherlopian, Ting Song, Qi Duan, Mathew A Neimark, Ming J Po, John K Gohagan, Andrew F Laine

**Affiliations:** 1 Physiology, Biophysics and Systems Biology Program, Weill Medical College of Cornell University, New York, NY, USA; 2 Department of Biomedical Engineering, Columbia University, New York, NY, USA; 3 Division of Cancer Prevention, National Cancer Institute, US National Institutes of Health, Bethesda, MD, USA; 4 Department of Radiology, Columbia University, New York, NY, USA

## Abstract

This paper presents a review of imaging techniques and of their utility in system biology. During the last decade systems biology has matured into a distinct field and imaging has been increasingly used to enable the interplay of experimental and theoretical biology. In this review, we describe and compare the roles of microscopy, ultrasound, CT (Computed Tomography), MRI (Magnetic Resonance Imaging), PET (Positron Emission Tomography), and molecular probes such as quantum dots and nanoshells in systems biology. As a unified application area among these different imaging techniques, examples in cancer targeting are highlighted.

## Systems biology

Systems biology [1-8] attempts to model the dynamics and structure of complete biological systems. To accomplish this goal, it enlists concepts and expertise from a wide array of fields such as mathematics, physics, engineering, and computer science in addition to the biological sciences. The "building blocks" of systems biology models are knowledge and data produced within experimental biology, and mathematical modeling provides the "cement" that links these "building blocks." Systems biology extensively uses computational technology and numerical techniques to simulate complex biological networks. The goal is not only to describe biology on a single component level, but also to understand system processes, mechanisms, and principles. The insight gained from simulation results can then be used to design *in vivo *and *in vitro *experiments, and in turn further develop models in an ever more refined description of physical and biological reality.

**Figure 1:**
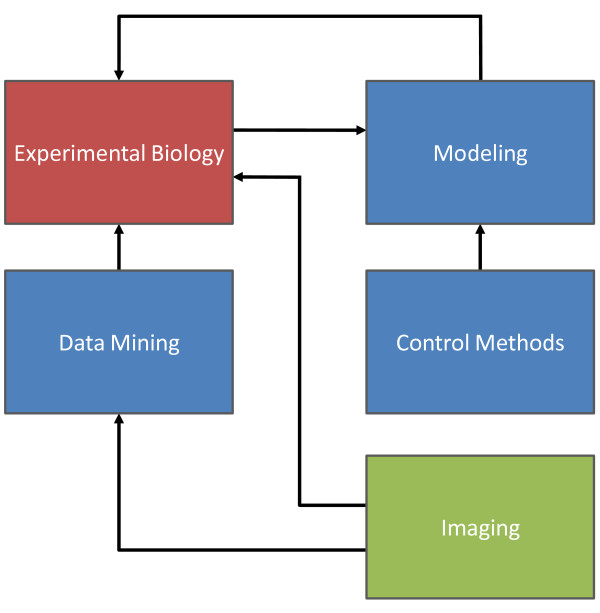
**Components of systems biology**. Systems biology supplements experimental biology by providing methods to both interpret and validate new findings. Data mining provides a way to gain insight from large data sets, while control methods facilitate the interplay of modeling and experimental biology. Imaging can be used for qualitative assessments during experiments and also provide a large amount of data amenable for data mining.

As seen in Figure 1, experimental biology can be aided by data mining, and thus statistical analysis, which can be used to extract hidden patterns from large quantities of data to form hypotheses. Hypothesis-driven models can then describe system dynamics. In this regard, systems biology includes *in silico *simulations in addition to *in vitro *and *in vivo *experiments. With adequate models of biological function it is possible to use control methods, as in incorporating feedback and regulatory loops into models and system understanding. Imaging plays a unique role in that it can both provide insight during experiments and also be used to gather data in a high throughput fashion for later analysis.

There are two approaches or "avenues" describing the interplay between experimental and theoretical biology. The traditional approach has been for experimental results to drive model creation. An alternative approach is to generate models based on first principles and then test model-inspired hypotheses by new experiments. The ideal situation is to traverse both "avenues," and so central to the methodology of systems biology is the notion of an iterative and strategic interplay between experimentation and modeling [1,3,5,6,8-11].

## Role of imaging

Since the time of Galileo, imaging has been the "eyes of science." Modern imaging technologies allow for visualization of multi-dimensional and multi-parameter data. Imaging is increasingly used to measure physical parameters such as concentration, tissue properties, and surface area [12] and to glean temporal insight on biological function. Molecular probes can also be employed to allow for both therapeutic and diagnostic applications [13-16]. As the spatial resolution and acquisition frequency of imaging techniques increase, using imaging to monitor substrate and protein dynamics in real time may be more readily achieved. Data acquired by imaging can provide the basis for mathematical modeling of protein kinetics and biochemical signaling networks [12,17]. Imaging can also be a suitable means to test computational models already developed.

Digital image processing techniques such as segmentation and registration contribute to model creation and validation strategy. Segmentation can help outline and identify particular regions in an imaged volume where there is biological activity of interest taking place. Registration can assist in the alignment of imaged volumes and areas acquired at different times. Segmentation and registration used together can generate time series data for validating systems biology models. After segmentation and registration, volume and surface rendering can be employed for data visualization [12]. Implementing systems biology models in conjunction with imaging provides a way to refine understanding of biological systems [18]. Eventually, as imaging tools become more widely used, and as more biological processes are understood, systems biology models can be developed that will have true predictive capabilities. To reach this end biology will be propelled by computational models, and imaging science will guide their formulation and validation.

## Cancer applications

Major efforts are underway to apply systems biology methods to oncology [19,20]. Increasingly sophisticated and accessible genomics, proteomics, and metabolomics high throughput experiments provide a basis for new types of oncology research [21]. The number of published results based on gene expression microarray data alone has increased by a factor of 1700% over the last decade [22]. These advances in experimental systems biology coupled with new analysis techniques and quantitative imaging software tools are helping to generate a more complete picture of many cancer related signaling pathways [21-23].

The actual development of cancer is a complex process, requiring the accumulation of multiple independent mutations each governing different pathways of cell growth and the cell cycle [21,24]. Genome-wide experiments have shown many signaling pathways to be interrelated and with many transcription factors serving as co-regulators in other signaling pathways [21,24,25]. This integrated nature of cancer pathways leads to difficulty in targeting specific pathway components. Efforts are underway to create comprehensive models of the cell cycle that can be used to better understand both the dynamics of cancer and to enable the design of targeted therapeutics [21,24,26].

Advances in molecular imaging can help to satisfy the post-genomic era need for the study of complete biological pathways, and this can potentially accelerate the achievement of a systems level understanding of biological complexity [27,28]. Molecular imaging enables the determination of both the temporal and the spatial distributions of biological processes throughout an intact living subject. With this approach, it is possible to obtain more meaningful results than can be achieved by comparable *in vitro *methods [29].

With the advance of molecular imaging techniques, properly tagged molecules can be visualized leading to insights on cell function, membrane binding sites, and the effectiveness of particular therapies [30-33]. For example, integrating imaging and modeling has led to successful monitoring of immune system functionality via T cell activity [10] and the development of bacteriophages for cancer targeting [34]. It is this type of integration of imaging and modeling that can enable new advances in oncology and other fields in the biomedical sciences.

## Imaging on multiple scales

The next generation of imaging tools will include innovative microscopy methods, ultrasound, CT (Computed Tomography), MRI (Magnetic Resonance Imaging), and PET (Positron Emission Tomography). In the coming years, improvements in temporal sampling and spatial resolution will certainly continue. With the advent of molecular probes, imaging can be conducted not only to visualize gross anatomical structures, but also to visualize substructures of cells and monitor molecule dynamics. Thus, the imaging modalities of microCT, microMRI, fMRI, MRS, microPET will also play important roles. A comparison of these imaging technologies is summarized in Table 1. As for the organization of this review paper, each imaging technique is profiled with its respective underlying principle, a description of selected current applications, and a discussion of advantages and known limitations. As a common application area, topics in cancer targeting are highlighted.

**Table 1:** Comparison of imaging technology for systems biology

Imaging Technique	Resolution References	Spatial Resolution	Scan Time	Contrast Agents and Molecular Probes	Key Use
Multi-photon Microscopy	[29,38]	15 – 1000 nm	Secs	Fluorescent proteins, dyes, rhodamine amide, quantum dots	Visualization of cell structures
Atomic Force Microscopy	[104]	10 – 20 nm	Mins	Intermolecular forces	Mapping cell surface
Electron Microscopy	[41]	~5 nm	Secs	Cyrofixation	Discerning protein structure
Ultrasound	[29]	50 μm	Secs	Microbubbles, nanoparticles	Vascular imaging
CT/MicroCT	[29,70]	12 – 50 μm	Mins	Iodine	Lung and bone tumor imaging
MRI/MicroMRI	[29,76]	4 – 100 μm	Mins – Hrs	Gadolinium, dysprosium, iron oxide particles	Anatomical imaging
fMRI	[105]	~1 mm	Secs – Mins	Oxygenated hemoglobin (HbO_2_) deoxygenated hemoglobin (Hb)	Functional imaging of brain activity
MRS	[106,107]	~2 mm	Secs	N-acetylaspartate (NAA), creatine, choline, citrate	Detection of metabolites
PET/MicroPET	[29,108]	1 – 2 mm	Mins	Fluorodeoxyglucose (FDG), ^18^F, ^11^C, ^15^O	Metabolic imaging

The various micro versions of the imaging modalities (MicroCT, MicroMRI, MicroPET) as well as the microscopy techniques (Fluorescence, Multi-photon, Atomic, Electron) are primarily used in either cellular or animal studies. The remaining modalities (Ultrasound, CT, MRI, MRS, PET) are more widely used clinically.

## Microscopy

### Basic principles

**Figure 2:**
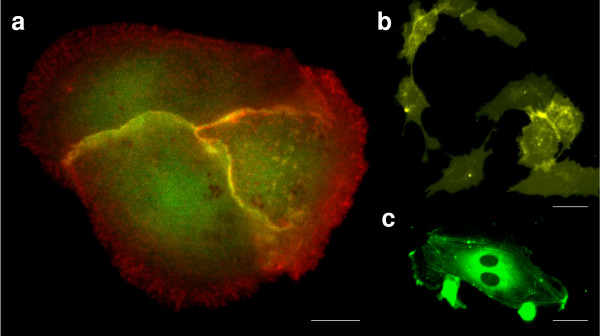
**Fluorescent protein applications**. (a) Three Madin-Darby canine kidney epithelial cells with GFP-rac1 and dsRed-E-cadherin. Rac1 is a pleiotropic signaling molecule that is closely associated with cell-cell adhesion and cell motility. E-cadherin is a cell-cell adhesion protein responsible for facilitating communication between two contacting cells. Scale bar: 10 μm. Contributed by Lance Kam (Columbia University, New York). (b) Membranes of human umbilical cord endothelial cells visualized using EYFP. Scale bar: 40 μm. (c) GFP-actin labeled human umbilical cord endothelial cell undergoing mitosis, with actin filaments aligned toward the centrioles. Scale bar: 30 μm. Contributed by Samuel Sia (Columbia University, New York).

**Figure 3:**
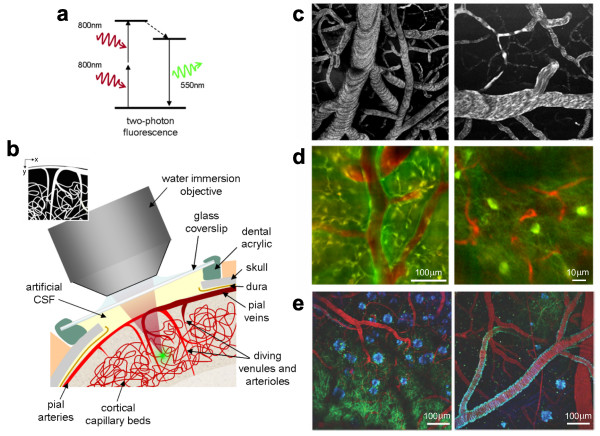
**Two-photon microscopy of *in vivo *brain function**. (a) Basic mechanism of two-photon fluorescence. (b) Schematic of surgical preparation of exposed cortex, with sealed glass window and microscope objective positioning. Green dot shows location of two-photon fluorescence. (c) Examples of two-photon maps of the vasculature following intravenous injection of dextran-conjugated fluorescein. Black dots and stripes show red blood cell motion. (d) Dual-channel imaging of neuronal (green) and vascular (red) signals: (left) Oregon Green 488 BAPTA-1 AM calcium sensitive dye stained neurons and (right) transgenic mouse expressing green fluorescent protein (GFP) in a subpopulation of neurons (mouse supplied by Jeffrey M. Friedman, Rockefeller University, New York) [101]. Texas dextran red is the intravascular tracer in both cases. (e) Three channel imaging of Tg2576 APP Alzheimer's disease mouse model with amyloid-targeting dye (blue), GFP expressing neurons and dendrites (green) and vasculature (red). Adapted from [52] and contributed by Elizabeth Hillman (Columbia University, New York).

The advent of fluorescence microscopy has been a major step forward in the study of living cells. Leveraging the characteristic emissions of excited biological fluorophores, such as fluorescent proteins, it is possible to gain insight on cell structure and function (Figure 2). Following traditional fluorescence microscopy has been the development of multi-photon methods, where fluorophores are excited by two or more photons [35]. Multi-photon absorption is achieved with a single pulsed laser focused to a diffraction-limited spot on the specimen. With higher peak power, there is an increase in probability for multi-photon absorption leading to fluorophore excitation. Two-photon fluorescence is depicted in Figure 3a. To meet the excitation energy in this case, two 800 nm photons are used. One 400 nm photon is of equivalent energy, as can be used in single photon excitation, but with multi-photon methods only the area of the laser focus on the specimen is excited. Due to more focused excitation, there is a lower overall phototoxic effect. Also, as scattering of longer wavelength photons is less, multi-photon methods have deeper penetration when compared to single photon excitation.

In STED (Stimulated Emission Depletion) microscopy [36,37], two pulsed lasers are used in tandem to break the diffraction barrier. The first laser pulse has a wavelength that excites fluorophores, and is immediately followed by a second laser pulse that depletes fluorescence. The fluorescence depletion is achieved as the wavelength of the second laser is tuned to be longer than the fluorescence emission. Absorption of a photon from the second laser induces electrons to drop to a lower energy level (stimulated emission) preventing typical fluorescence. The difference in area of the two focused beams leaves only a very small area from where fluorescence is detected. This area is smaller than a diffraction-limited spot. Using STED, images have been captured with a resolution of ~30 nm [35]. In another study using rhomadine amide and a photoswitching technique, a resolution of 15 nm was achieved [38]. In [39,40], an optical trapping system was used to make angstrom resolution measurements of pair based stepping of RNA polymerase, and thus has established an important resolution benchmark in molecular biology.

Electron microscopy has offered a resolution of ~5 nm for imaging biological tissue [41]. However, to prepare a sample to be imaged by an electron microscope is a rigorous process that does not allow for imaging of live samples [42]. One common sample preparation technique is cryofixation, which is a high pressure and deep freezing technique that results in contrast in electron microscopy [43]. Even though the samples are no longer viable, electron microscopy has provided invaluable insights on the structural details of organelles and membranes [44].

Atomic force microscopes do not acquire information optically, but rather by recording intermolecular forces between a probe tip and a surface. The primary information acquisition component of an atomic force microscope is a cantilever with a nanometer-scale silicon tip. The tip is brought in close proximity with the sample and the deflection of the cantilever due to Van der Waals forces is recorded to generate a contour map of the sample surface [45,46]. In comparison with an electron microscope, the sample does not need any special treatment that would actually destroy the sample and prevent its reuse. However, using contact or tapping mode of an atomic force microscope, which impinges on the sample surface to acquire measurements such as strain, can mechanically damage cells and tissue. Other probe microscopy techniques include scanning tunneling microscopy and near-field scanning optical microscopy [47].

### Current applications

Fluorescence microscopy is often used in systems biology and there is a strong push for the development of high throughput methods. In the application of genome-wide RNAi screens to document the phenotype for each suppressed gene [48,49], there can be millions of images from a single screen which can amount to several terabytes of data [50]. It is systems biology modeling that relieves the bottleneck of processing this large amount of RNAi screen image data by providing an efficient means of classification. With high throughput microscopy there is much more data generated than can be annotated or evaluated manually, and so developing a fine-tuned and efficient classification model is paramount for unlocking the potential of high throughput methods.

As seen in Figure 2, fluorescent proteins can be used to visualize many functional and structural aspects of cells. Using multi-photon methods as well can provide insight on cell structural and biochemical changes [51]. Multi-photon methods have a wide array of applications including *in vivo *brain imaging in animals (Figure 3) [52-54], where cortical micro-architecture has been investigated with single cell resolution [55]. Electron microscopes have been used to elucidate macromolecule structure [41]. As for cancer applications, atomic force microscopes have been used to monitor the super-coiled state of DNA, which is preferential to the binding of the tumor suppressing protein p53 [56]. Also in regard to detecting levels of p53 in cells, fluorescence microscopy has been used to determine the effectiveness of oncolytic adenoviruses. Specially designed oncolytic adenoviruses target cancerous tissues and are programmed to replicate if the cellular p53 level is low. Viral oncolytic therapy is an intense research area for cancer treatment and as microscopy techniques advance so will the ability to assess the effectiveness of viral vectors for tumor ablation [57,58].

### Advantages and limitations

It has been long held that the wave nature of light imposes a seemingly fundamental limit on the resolving power of a microscope. The limitation was approximately half the wavelength of visible light or 200 nm. Recently, there has been over a 10-fold resolution improvement with advances in microscopy [35,38]. However, optical techniques have limited penetration as light readily scatters in tissue. This can be partially ameliorated by using more powerful lasers, but this in turn can lead to increased photobleaching effects which can limit the amount of time that an experiment can run.

Microscopy, as with other modern imaging techniques, has become ever more dependent on software for image acquisition and analysis. Imaging technology can be enhanced or limited by the software it is coupled with. Table 2 contains an overview of current microscopy image analysis software. With advances in acquisition algorithms and optics holographic microscopy has been achieved, by which full three-dimensional information can be acquired in a single image [59,60]. As a result, volumetric time series data can be collected without the need of changing focus and scanning multiple z-planes.

**Table 2:** Overview of microscopy image analysis software

Vendor	Package Name	Image Support	Supported Devices	Website
MVIA	Image Analysis Software	2D/3D	A	
Clemex	Clemex Vision PE	2D/3D	A	
MIS	Pax-It PI-M300A	2D	E	
Media Cybernetics	Image-Pro Bundled Solutions Image-Pro AMS Image-Pro MDA Image-Pro MC Image-Pro 3D Suite	2D/3D	A, D	
iMTtechnology	iSolution DT	2D/3D	n/a	
Dewinter Optical	Dewinter Caliper Pro Dewinter Biowizard Dewinter Material Plus Dewinter Foundry Plus Dewinter Micro Measurement Pro	2D	E	
MBF BioScience MicroBrightField	AutoNeuron Confocal SD ImageStackModule NeuroLucida SolidModelingModule SteroInvestigator VirtualSliceModule	2D/3D	C, D, E	
Nascent Technology	MedicalPlus MeasurePro CapturePro	2D	A	
Intelligent Perception	Pixcavator Image Analyzer	2D	n/a	
GSA Bansemer & Scheel GbR	GSA Image Analyser	2D	n/a	
Broad Institute	CellProfiler* CellVisualizer*	2D	n/a	
IMAS	CellObserver EliSpot Process Analysis	2D	A, D, E	
Wadsworth Center	Spider*	2D/3D	E	
MCID	MCID Core	2D/3D	A, E	
ImageJ	ImageJ for Microscopy*	2D/3D	C, D	
Scion Corporation	Scion Imaging Software*	n/a	n/a	
(*) Open source/freeware software packages A = Automated microscope B = Planar microscope C = Confocal microscope D = Functional microscope E = Digital microscope				

Gaining insight from microscopy images typically requires some level of processing and analysis. Both commercial and open source software packages are available that can supplement or totally drive microscope usage.

## Ultrasound

### Basic principles

Ultrasound imaging entails moving a hand held probe over the patient and using a water-based gel to ensure good acoustic coupling. The probe contains one or more acoustic transducers and sends pulses of sound into the patient. Whenever a sound wave encounters a material with different acoustical impedance, part of the sound wave is reflected which the probe detects as an echo. The time it takes for the echo to travel back to the probe is measured and used to calculate the depth of the tissue interface causing the echo. The greater the difference between acoustic impedances, the larger the echo is. A computer is then used to interpret these echo waveforms to construct an image [61].

### Current applications

**Figure 4:**
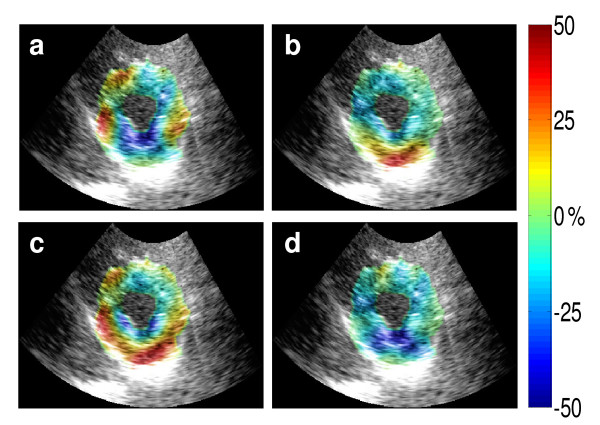
**Transthoracic echocardiography and elastography of a healthy human left ventricle**. (a), (b), (c), and (d) are the lateral, axial, radial, and circumferential systolic strains from myocardial elastography between end diastole and end systole, respectively. Strains are displayed on a scale of ± 50%. All the images were acquired approximately at the papillary muscle level and shown at end systole. Contributed by Elisa Konofagou (Columbia University, New York).

Ultrasound has had a tremendous impact in cardiology. As seen in Figure 4, the use of ultrasound can enable the coupling of anatomical and strain information of the heart [62]. Going from the organ level to the molecular level has been made possible by advances in microbubble manufacturing. Microbubbles themselves are several micrometers in diameter and are intravascular tracers [63]. Ligands can be attached to microbubbles to make them target specific [64]. Many clinical applications of contrast enhanced ultrasound, such as monitoring angiogenesis and inflammatory response, rely on ultrasound detection of microbubbles that contain gas. Since microbubbles are confined to the vascular space, they are useful for targeting antigens expressed on endothelial and blood cell surfaces [63]. Smaller nanoparticle based contrast agents are also available that are capable of extravascular migration in regions of vascular injury or regions where vascular permeability is abnormally high. Ultrasound can also cause a mechanical interaction with microbubbles, leading to their destruction and the subsequent release of therapeutic compounds [65].

In tumor and angiogenesis models, surface expression of *α*_*v*_*β*_*3 *_has been demonstrated to be a strong ligand for targeting endothelial cells in angiogenic vessels [63]. In order to image this surface expression, microbubbles were conjugated with peptides that bind to *α*_*v*_*β*_*3*_. These microbubbles have been shown to have a binding preference to the endothelial surface of Fibroblast Growth Factor (FGF) stimulated neovessels. The extent of neovascularization in a matrigel model matched the image enhancement in ultrasound images to a large extent. Thus, ultrasound imaging served to help validate this experimental model for angiogenesis [63].

### Advantages and limitations

The signal-to-noise ratio for ultrasound images is much lower with nanoparticles than those using microbubbles. Although microbubbles are restricted to the vascular space, this can be an advantage since it minimizes potential signal interference from nonvascular cells [63]. As with other molecular imaging techniques, there is an inverse relationship between sensitivity and resolution for contrast enhanced ultrasound. The relative rate of unbound tracer clearance is also an important issue that determines temporal resolution. In this regard, with clearance time within minutes, microbubble tracers are ideal [63].

## CT/MicroCT

### Basic principles

Intrinsic differences in X-ray absorption among water, bone, fat, and air provide contrast in Computed Tomography (CT). In CT, a low energy X-ray source and a detector rotate around the subject, acquiring volumetric data. The detectors are typically Charged Coupled Devices (CCD) and act to phototransduce incoming X-rays [66]. For animal studies, microCT machines can be used which typically operate with higher energy X-rays when compared to human scanners. The increase in energy improves resolution, but exposes the specimen to more ionizing radiation which has adverse health effects.

### Current applications

CT has relatively low soft tissue contrast for tumors and surrounding tissue, but with iodinated contrast agents organs and tumors can be detected [29]. As a result, incorporating iodine into new probes for CT imaging may be necessary. Furthermore, to detect a tumor or other target there must be sufficient site-specific accumulation of probes to result in attenuation of X-rays. With differential attenuation of X-rays, the target can be more readily delineated [7].

CT can be used to image lung tumors and bone metastasis, given its fast imaging time and high spatial resolution. High throughput techniques using microCT have been used for phenotyping large numbers of transgenic mice and detecting macroscopic abnormalities [64]. In [67], the co-registration of microCT images containing tumor structural details with bioluminescence images allowed for the study of cell trafficking, tumor growth, and response to therapy *in vivo*. This image analysis method could potentially be used for assessing hematological reconstitution following bone marrow transplantation.

**Figure 5:**
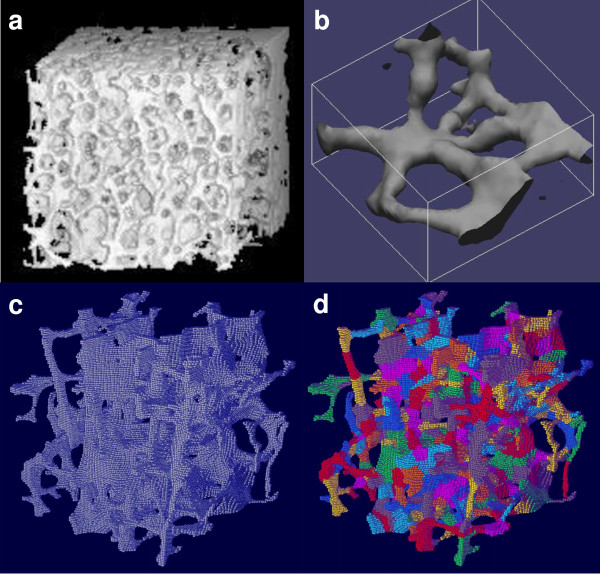
**Complete volumetric decomposition procedure on a vertebral trabecular bone sample**. (a) Example microCT bone volumetric data. (b) Closer view of plate and rod microstructures. (c) MicroCT image of a trabecular bone sample. (d) Completely decomposed trabecular bone structures with individual trabeculae labeled by color for each voxel. Image volume: 5 mm^3^. Contributed by X. Edward Guo (Columbia University, New York).

As seen in Figure 5, microCT imaging and volumetric decomposition were used to provide insight on trabecular bone microarchitecture [68]. The bone samples were decomposed into individual plates and rods, and this imaging and processing scheme has been successfully applied to anatomic sites such as the proximal femur, proximal tibia, and spine. Several key morphological features of trabecular bone architecture were studied: plate and rod size, thickness, number density, and orientation. With this level of detail, it was determined that trabecular plates play an essential role in determining the elastic properties of trabecular bone [68]. Assessing such properties can be important for gauging bone health in conditions such as osteoporosis, and for designing viable replacement tissue in tissue engineering applications [69].

### Advantages and limitations

A key advantage of CT is its high spatial resolution, 12 – 50 μm [29,70], which is needed to visualize fine anatomical details. CT can also be combined with functional imaging technologies that provide dynamic and metabolic information. The radiation dose of CT, however, is not negligible and this limits repeated imaging in human studies due to health risks [64].

## MRI/MicroMRI, fMRI, and MRS

### Basic principles

Magnetic Resonance Imaging (MRI) is achieved by placing a subject in a strong magnetic field, typically 1.5 or 3 Tesla for human scanners, which aligns the hydrogen nuclei spins in a direction parallel to the field. A Radio Frequency (RF) pulse is applied to the sample which causes the spins to acquire enough energy to tilt and precess, where an RF receiver can record the resulting signal [71]. After the removal of the RF pulse, the spins realign parallel to the main magnetic field with a time constant of T1 which is tissue dependent. Signal strength decreases in time with a loss of phase coherence of the spins. This decrease occurs at a time constant T2 which is always less than T1. Magnetic gradients are used to localize spins in space, enabling an image to be formed. The difference in spin density among different tissues in a heterogeneous specimen enables the excellent tissue contrast of MRI [71]. MicroMRI follows the same principles, but a much higher magnetic field strength is used for animal studies. Increasing magnetic field strength improves resolution, but can disturb the visual system and lead to peripheral nerve stimulation.

Functional Magnetic Resonance Imaging (fMRI) is a modality used to image brain activity in response to specified stimuli. When a stimulus solicits a response from a certain area of the brain, metabolism in that region increases. Metabolic demand leads to an increase in blood flow and more oxygenated hemoglobin in the region. As the supply of oxygenated hemoglobin exceeds the metabolic demand, the concentration of oxygenated hemoglobin increases. The balance between oxygenated and deoxygenated hemoglobin is altered leading to a change in image contrast. To detect a change, the image is compared with baseline measurements. Typical cortical activation leads to a 1 – 5% increase in image intensity [72].

**Figure 6:**
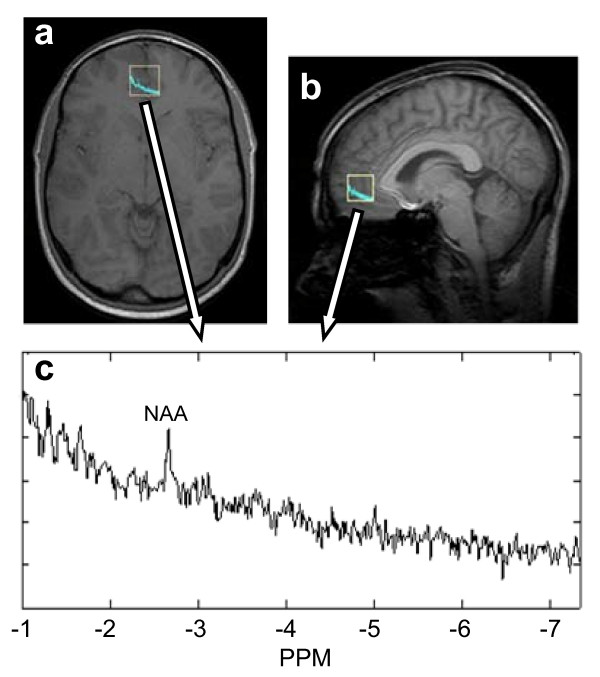
***In vivo *point resolved (single voxel) MRI spectroscropy**. (a) Axial and (b) sagital views of human brain and outlined voxel for MRS. (c) ^1^H spectrum with readily visible N-acetylaspartate (NAA) peak. An aberrant NAA peak can be an indicator of brain injury or disease.

Magnetic Resonance Spectroscopy (MRS) is an emerging imaging and biochemical analysis technique in biomedical science. It combines the analytical ability of Nuclear Magnetic Resonance (NMR) to identify biochemical species with the capabilities of MRI to isolate individual voxels which are three-dimensional pixels. MRS employs chemical shift imaging to localize spectra for individual voxels [73]. This is achieved by phase modulated RF pulses which eliminate signal contamination into neighboring voxels. When MRS is combined with MRI, concurrent anatomical and biochemical information is obtained (Figure 6).

### Current applications

The range of microMRI applications spans from purely experimental to preclinical. MicroMRI technology has been used to track stem cells, monitor immune cell proliferation, and describe embryological development [74]. It has also been used to obtain three-dimensional high resolution representations of bone structure [75]. MicroMRI has advanced to the point at which individual cells, and their organelles, can be imaged with spatial resolution of <4 microns. Images of a *paramecium *and a *spirogyra alga *were acquired utilizing a magnetic field of 9 Tesla, phase encoding in all three axes (which improves signal to noise), and Carr-Purcell echo refocusing (incorporation of multiple 90 degree spin echo pulses into the sequence to minimize signal loss due to sample inhomogeneity) [76].

Contrast agents have been developed with greater affinity for cellular and molecular targets. These include iron oxide particles (which have been used to label individual T cells), manganese ions (which act as a paramagnetic surrogate of calcium), and caged compounds. The latter involves chelated gadolinium surrounded by an enzyme substrate, which physically obstructs water molecules from approaching the gadolinium. When an enzyme cleaves the substrate, water is able to approach the gadolinium. This in turn reduces T1 and increases contrast. The caged-compound technique has been used to demonstrate regionalized *in vivo *gene expression in frog embryos whereas manganese ions have been used to trace neuronal pathways [74].

fMRI is used to study the functions of the living brain in a non-invasive manner. It has been shown with fMRI that different cognitive functions, such as attention, perception, imagery, language, and memory, elicit specific cognitive activation patterns in different regions of the brain. One common clinical use of fMRI is in the treatment of patients with brain tumors, and a primary treatment goal is to preserve functional brain tissue. fMRI is used to determine the functionality of brain tissue surrounding the tumor so that potentially harmful therapy can be directed away from critical areas [77].

Due to the ability of MRS to identify the presence of molecules within voxels, many studies have been devoted to using it to help diagnose cancer and characterize neoplastic tissue. Currently, MRS has been successfully employed in regard to brain, breast, and prostate cancer through identification of various biochemical markers of neoplasm in the imaged volume [78,79]. ^1^H has been the element of choice because of its large abundance, but studies involving ^31^P and ^13^C appear promising. The latter has been used as an effective dynamic marker of metabolic processes through a hyperpolarization technique [80].

### Advantages and limitations

The two chief advantages of MRI are its excellent tissue contrast and lack of ionizing radiation [74]. Improved signal-to-noise ratio and resolution can be obtained via a small receiver coil radius and high magnetic field strength. However, high magnetic field strength is problematic in human applications because of arising physiological effects such as nausea and visual abnormalities. Also, higher field strength leads to other technical challenges including an increase in the operating frequency, which potentially generates artifacts.

The main advantage of fMRI is its ability to non-invasively image brain. Since image contrast is achieved through the levels of oxygenated and deoxygenated hemoglobin, no external contrast agent is needed. However, due to the faster temporal resolution needed to acquire images of dynamic brain activity, spatial resolution is reduced.

Due to the ability of MRS to reveal the presence of particular biomedical molecules and compounds within an *in vivo *sample, it seems ideally poised for use in systems biology research. However, certain challenges must be overcome such as large voxel size, long sampling times, and questionable quantitative accuracy of assessing molecular concentrations [81].

## PET/MicroPET

### Basic principles

**Figure 7:**
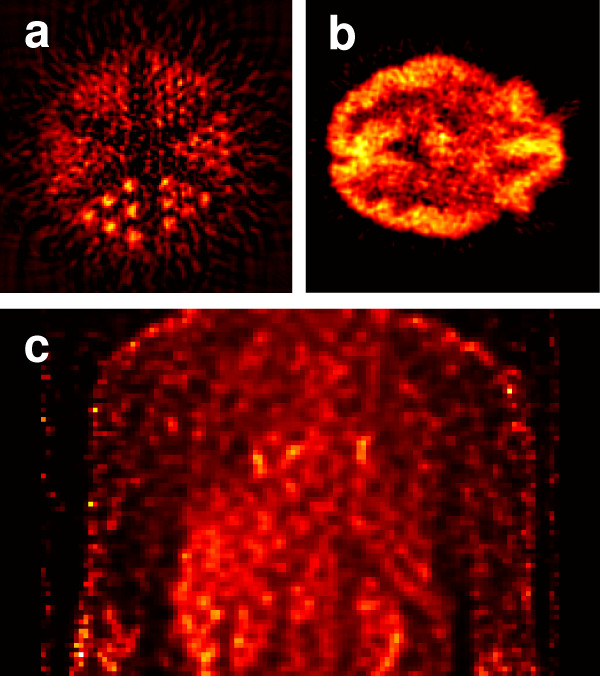
**Phantom and anatomical PET images**. (a) ^11^C PET image of a rod phantom. (b) FDG PET image of a brain. (c) Coronal view of thoracic area from a whole body PET scan.

In Positron Emission Tomography (PET), radioactive tracers are incorporated into metabolically active molecules and then injected intravenously. There is a waiting period while the metabolically active molecules are concentrated in the target tissue. The molecule most commonly used in PET is fluorodeoxyglucose (FDG), which has radioactive fluorine and is readily taken up by tumors. The radioactive tracer decays and produces two 511 keV gamma-rays, which result from the annihilation of a positron and an electron. The two resultant gamma-rays are emitted nearly 180 degrees apart and observed by detector rings. Figure 7 contains several sample PET images. The sensitivity of PET at detecting molecular species is relatively high, in the range of 10^-11 ^– 10^-12 ^M. For animal studies, microPET has a volumetric resolution of 8 mm^3^, while next generation scanners have over an 8-fold increase in resolution and a field of view that encompasses the whole body of a mouse [29,82].

### Current applications

There are many radioactive tracers for PET that are used in different preclinical and clinical applications [7]. The tracers that target specific tumors are essential for systems biology studies due to the information provided regarding metabolic activity [83,84]. Examples of small targeting ligands include ^11^C-labelled N-methylspiperone and ^18^F-labelled spiperone for targeting dopamine receptors on pituitary adenomas [83].

PET is useful in systems biology studies related to bone metabolism [85] and metastasis. Bone metastasis is common for several cancers, including prostate, breast, and lung [86]. ^15^O-labelled water can be extracted from the blood and used to assess tumor blood perfusion. Tumors are in constant need of nutrients from the blood, and tumor neovascularization provides a crucial lifeline for rapidly dividing tumor cells. The uptake of tracer into tissues is proportional to delivery, and so is a measure of perfusion [87].

PET can be used for measuring therapeutic effects on disease processes. Specific metabolic enzymes that are selectively expressed in prostate cancer cells constitute such a target. In [11], genes that were differentially expressed between early stage and late stage prostate cancer were studied. L-lactate dehydrogenase-A catalyzes the formation of pyruvate from S-lactase and was expressed at a high level in the late stage cancer cells. PET tracers based on this process would serve to validate this finding and may allow for the identification of prostate cancer metastasis [11].

### Advantages and limitations

PET is a highly sensitive, minimally-invasive technology that is ideally suited for pre-clinical and clinical imaging of cancer biology. By using radioactive tracers, three-dimensional images can be reconstructed to show the concentration and locations of metabolic molecules of interest [2]. Since the study of cancer cells in their normal environment within intact living subjects is essential, PET is ideally suited for monitoring molecular events early in the course of a disease, as well as during pharmacological or radiation therapy. Furthermore, it can be used to acquire prognostic information and to image for disease recurrence [2,82].

PET spatial resolution is comparatively poor, and is limited by pixel sampling rate, the source size, and blurring in the phosphor screens of the detector rings. Another limitation of PET is that radioisotopes with very short half lives must be immediately injected after production. Due to the same decay type of the different radioactive tracers, it is only possible to trace one molecular species in a given imaging experiment or clinical scan [64].

## Molecular probes

Achieving contrast is essential to imaging technology and is often made possible by contrast agents or molecular probes. As mentioned above, fluorescent proteins have played a key role in microscopy studies providing insight on cell structure. Microbubbles have greatly enhanced the use of ultrasound both in imaging and therapeutic applications. For CT, iodine has been instrumental in differentiating tissue types. In MRI based technologies, manipulation of hydrogen spins has allowed for excellent soft tissue contrast and functional imaging of the brain. FDG and other radioactively labeled tracers have enabled targeting of cancer and imaging of metabolic activity with PET. Below, two promising molecular probes are profiled, quantum dots and nanoshells, which may yield a new array of imaging applications.

## Quantum dots

### Basic principles

Quantum dots (QD) are a class of polymer-encapsulated and bioconjugated probes that can fluoresce at multiple wavelengths spanning the visible spectrum. Larger quantum dots emit red light while smaller ones emit blue light. Quantum dots themselves are comprised of a semiconductor core, encased in another semiconductor material that has a larger spectral band gap. This construction enables fluorescence upon excitation. Quantum dots can be packaged in amphiphilic polymers and conjugated with targeting ligands for imaging applications [88]. Under harsh conditions such as wide pH range (1–14), varied salt conditions (0.01 to 1 M), and a strong corrosive environment (1.0 M hydrochloric acid), quantum dots demonstrate extraordinary resiliency and sustained functionality [14].

### Current applications

**Figure 8:**
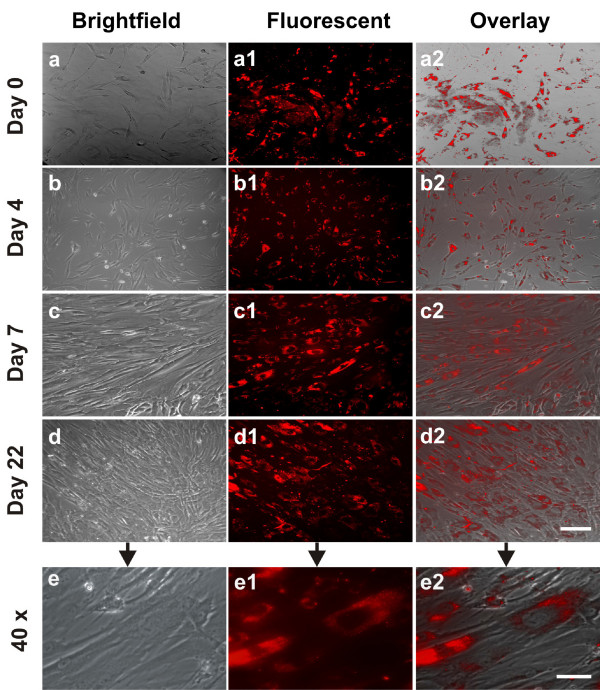
**Quantum dot labeled human mesenchymal stem cells undergoing proliferation**. hMSCs were incubated for 16 hrs in a 30 nM solution of bioconjugated QDs (a-a2). Following the removal of extracellular QDs, QD-labeled hMSCs and unlabeled hMSCs of the same subpopulation were continuously cultured for 4, 7 and 22 days (b-b2, c-c2, d-d2, respectively). Scale bar: 30 μm. QDs were internalized in the cytoplasm, even after 22 days of culture-expansion (e-e2), apparently endocytosed as aggregates. Scale bar: 5 μm. Reproduced from [89] and contributed by Jeremy Mao (Columbia University, New York).

Figure 8 shows the proliferation of human Mesenchymal Stem Cells (hMSC) that are labeled with quantum dots. After 22 days the quantum dots remained incorporated in the hMSCs. This study suggests that bioconjugated quantum dots are a viable probe for long-term labeling of stem cells [89].

Ligands on quantum dots can be tailored to target specific cancer lines. Quantum dots fashioned to target prostate cancer, QD-PSMA (Prostate Specific Membrane Antigen), showed active emission in the presence of C4-2 prostate cancer cells while other quantum dots did not [14]. Quantum dots can also be used to passively target tumors since leaky tumor vasculatures retain more quantum dots than surrounding healthy tissue. Thus, by both active binding and passive diffusion, more quantum dots will be present near cancerous tissue [14]. With the targeting capabilities of quantum dots there is potential for use as a delivery vehicle for therapeutic compounds. Delivery schemes can be based on the release of a therapeutic compound triggered by ligand binding [13,14,16,90]. As an example of a drug delivery application, in [91] quantum dots with cadmium sulfide were used as chemically removable caps inside mesoporous silica nanospheres to prevent the premature release of drug molecules. Targeted release of drug molecules was mediated by disulfide bond-reducing agents. Quantum dots could also be used in photodynamic therapy by which there is an energy transfer from the quantum dots to target cells, leading to the generation of reactive oxygen species, and thus potentially inducing apoptosis [92,93]. One limitation of such a therapy *in vivo *would be reliable and localized energy transfer to ensure the destruction of specific cells.

As applied to bacteriophage development, quantum dots can be multi-purpose by validating the design model as well as showing the effectiveness of tumor targeting [34]. One design model for bacteriophages with quantum dots is based upon the characteristics of quantum dots themselves. These are namely durability due to the co-polymer shell and flexibility due to the possibility of several different ligands. Experiments have been conducted with quantum dot embedded bacteriophages in both *in vitro *and *in vivo *with the goal of destroying cancerous tissues. Iteratively designing and creating bacteriophages is an example where quantum dots provide both the effective targeting means, but also the validation of the design model due to visualization of ligand binding [34].

### Advantages and limitations

Information acquired by using quantum dots are constrained by the physical limits of fluorescence microscopy, since that is the imaging technique typically used when detecting emissions from quantum dots. There have been some studies using quantum dots in electron microscopy, which has an order of magnitude higher resolution than light microscopy [90]. The quantum dots themselves experience "blinking," as in each quantum dot randomly switches from on to off. Fortunately, the fluorescence of a bound quantum dot is stronger than of an unbound quantum dot. Still, the randomness of "blinking" imposes some limitations on applications requiring single molecule detection as well as on applications requiring quantification of total fluorescence [13]. Using two different color quantum dots, single molecule imaging has been achieved by co-localization on target molecules [94].

## Nanoshells

### Basic principles

**Figure 9:**
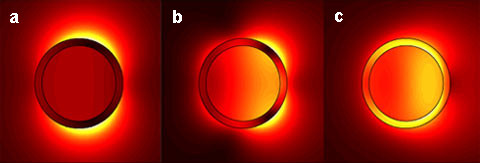
**Near field images of an Ag nanoshell**. Nanoshell exposed to (a) 721 nm, (b) 492 nm, and (c) 336 nm laser beam and consequential dipole, quadrupole, and dark plasmons, respectively. Surface plasmon oscillations are collective electron motion resultant of optical illumination, and subsequent modes are shown. Adapted from [102].

Nanoshells are a class of metal nanostructures consisting of a dielectric silica core surrounded by a very thin metallic shell. By varying the core to shell ratio and the overall size of the nanoshells, strong scattering properties can be achieved that result in resonance wavelengths generating heat [15]. See Figure 9 for cross sectional views of a nanoshell. Fabricating nanoshells with specific antibodies provide a means for scattering based molecular imaging [15,95], which provides molecule specific contrast on the nanometer scale.

### Current applications

**Figure 10:**
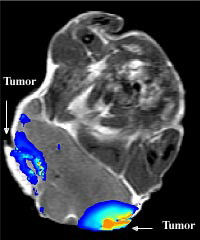
**Ablation of two tumors in a mouse**. With exposure to an external infrared laser source, the nanoshells resonate and thermally destroy tumor cells and their respective vasculature. Adapted from [103].

As seen in Figure 10, nanoshells can facilitate tumor ablation. In another cancer related study [15], nanoshells were used as contrast enhancers to image HER2 expression, a clinically relevant marker in human breast adenocarcinoma cells. Gold nanoshells were fabricated and tuned for Near Infrared (NIR) imaging. Then, the nanoshells were exposed to HER2 (specific) or IgG PEG-ylated (non-specific) antibodies to facilitate targeting of cultured human mammary adenocarcinoma cells. A microscope equipped with a bright field and dark field were used to evaluate cell viability. *In vitro *photothermal nanoshell therapy was performed and silver staining demonstrated the tissue targeting specificity of HER2 nanoshells as well as the non-specificity of IgG nanoshells. In addition to mediating photothermal destruction of breast cancer cells *in vitro*, it was demonstrated that NIR absorbing nanoshell bioconjugates can provide molecular specific optical contrast enhancement without cytotoxicity [15].

### Advantages and limitations

Optical imaging with nanoshells offers the potential for non-invasive, high resolution *in vivo *imaging at relatively low cost [15]. Scattering based optical imaging technologies rely on inherent changes in indices of refraction. Strategies that depend only on the intrinsic optical contrast within tissue have proved clinically valuable in some screening applications. However, such techniques are not sensitive enough to resolve an image based on disease biomarkers [15]. In cancer, when early detection is critical to reducing morbidity and mortality, the use of molecule specific contrast agents provides the ability to optically sense and image abnormalities long before pathologic changes occur at the anatomic level [15]. In the future, nanoshells may provide excellent contrast for other imaging modalities such as CT [96].

## Conclusion

In this review we have assessed a range of imaging techniques in systems biology spanning from microscopy to clinical imaging. In addition to the techniques reviewed, there are multiple other technologies that have lead to significant contributions to a systems level understanding of biological processes. Two such techniques are optical coherence tomography [97,98] and hyperspectral imaging [99,100]. With the refinement of current technologies and the development of new techniques, additional information will be available to help dissect biological systems.

**Figure 11:**
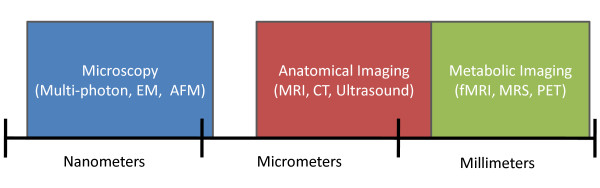
**Resolution spectrum of imaging techniques**. The schematic shows the resolution gap between microscopy and anatomical imaging. Metabolic imaging has successfully been linked to anatomical imaging despite having lower resolution. The schematic axis is linear.

As seen in Figure 11, there is a resolution gap between microscopy and anatomical imaging. This gap also represents the divide between experimental and clinical imaging applications. In contrast, acquiring anatomical and metabolic information with clinical scanners has been achieved by coupling imaging technologies. For example, it is now commonplace for usage of combination PET/CT scanners. This allows for metabolic information acquired in PET to be readily registered with higher resolution anatomical CT images. Also, with fMRI a slower MRI scan is also conducted to form a detailed brain atlas, to which functional images are later registered. As a result there is a resolution continuum between anatomical and metabolic imaging. To reach the same end for microscopy and anatomical imaging, molecular probes such as quantum dots and nanoshells may find more clinical applications and thus improve the resolution that can be achieved with clinical scanners.

Beyond improvements in resolution, a grand challenge remains for the imaging technology development community: to enable dynamic imaging of both biological system components and of their respective connections. For example, the ability to resolve and monitor an entire mammalian cortical circuit *in vivo *has yet to be realized. Electrophysiology has been increasing complemented by fMRI over the last 15 years, but with fMRI information on neural activity is provided as an indirect measure and on the scale of hundreds of thousands or millions of neurons. Two-photon imaging has provided for single cell resolution, but functionally visualizing hundreds of synapses performing computations is limited by axonal labeling of neuronal populations and also by overall temporal acquisition frequency. As a result, innovations in methods for visualizing neural circuitry and for deciphering spike times will be necessary to further advance systems neuroscience with imaging. In a broader set of application areas, using imaging to simultaneously monitor components of a molecular network will be useful in further understanding cellular processes, such as apoptosis which is critical for the development of new cancer treatments.

The further development of imaging technologies will continue to be important in the advancement of systems biology. Imaging can provide a wide array of data that can be used to build and validate models. The information acquired with imaging can be readily incorporated into models as biochemical concentrations, functional activity, and anatomical coordinates. In addition, imaging provides data for new discoveries and diagnostic information. Oncology and other areas in the biomedical sciences will benefit greatly from imaging and systems biology approaches.

## Authors' contributions

The authors collectively wrote this review.
